# Investigation on the Reaction Energy, Dynamic Mechanical Behaviors, and Impact-Induced Reaction Characteristics of PTFE/Al with Different TiH_2_ Percentages

**DOI:** 10.3390/ma11102008

**Published:** 2018-10-17

**Authors:** Zhongshen Yu, Xiang Fang, Yuchun Li, Jiaxiang Wu, Shuangzhang Wu, Jun Zhang, Junkai Ren, Mingshou Zhong, Liping Chen, Miao Yao

**Affiliations:** 1 College of Field Engineering, PLA Army Engineering University, Nanjing 210007, China; zhongshenyumail@sina.com (Z.Y.); fangxiang3579@163.com (X.F.); shsnake@163.com (S.W.); zhangjun19941106@sina.com (J.Z.); darkskyr@163.com (J.R.); zhongms7@126.com (M.Z.); yaoshunmiao@126.com (M.Y.); 2 School of Chemical Engineering, Nanjing University of Science & Technology, Nanjing 210094, China; clp319@njust.edu.cn

**Keywords:** PTFE/Al/TiH_2_ composites, reaction energy, mechanical behaviors, reaction characteristics, impact sensitivity

## Abstract

As a novel energetic material with quite a high energy density, titanium hydride (TiH_2_) was introduced into a polytetrafluoroethylene/aluminum (PTFE/Al) reactive material system for the first time. The effects of TiH_2_ on the reaction energy, dynamic mechanical responses, and reaction properties of the composites were investigated through adiabatic bomb calorimeter, split-Hopkinson pressure bar, and drop-weight experiments. The results show that the reaction heat of the composites improved significantly as the content of TiH_2_ increased. Under dynamic compression, these composites show obvious strain hardening and strain rate hardening effects. Besides, a certain amount of TiH_2_ granules helps to improve the material’s compressive strength, and the maximum would even reach 173.2 MPa with 5% TiH_2_ percentage, 10.1% higher than that of PTFE/Al. Mesoscale images of the samples after dynamic compression indicate that interface debonding between the particles and PTFE matrix and the fracture of the PTFE matrix are the two major mechanisms resulting in the material’s failure. In addition, the drop-weight experiments indicate that the material’s impact sensitivities are sensitive to the content of TiH_2_, which would be increased to within 20% of the content of TiH_2_ compared with PTFE/Al, and the reaction degree is also improved to within 10% of the content of TiH_2_. The retrieved reaction residues after drop-weight experiments imply that the reaction is initiated at the edges of the samples, indicating a shear-induced initiation mechanism of this kind of reactive material.

## 1. Introduction

Metal/fluoropolymer composites are normally considered to be inert and not sensitive to friction, heat, light, or shockwaves. However, when subjected to high-speed impact loading, these types of materials generate a violent exothermic reaction and release a tremendous amount of energy, which is even more than some high explosives such as trinitrotoluene (TNT) and cyclotrimethylenetrinitramine (RDX) [1]. Generally, compared with traditional energetic materials, metal/fluoropolymer composites have relative high mechanical strength after cold-pressing and sinter-hardening treatments. Due to the mechanical and energy characteristics, metal/fluoropolymer composites have been widely applied in both military and civilian application domains; for instance, they can be made into structural materials such as shaped charge liners and energetic fragments, or added into propellants/explosives as high-energy additives [2,3].

A polytetrafluoroethylene (PTFE) and aluminum (Al) mixture is one of the typical granular reactive materials, which undergo a violent exothermic reaction between Al and fluorine released from PTFE upon high impact loading. When the stoichiometric mass fraction of PTFE/Al is set at 73.5/26.5 for a complete reaction, the two components will react according to the following chemical equation [4]:

4Al + 3(–C_2_F_4_–) → 4AlF_3_ + 6C + 8.67 MJ/kg (1)

As is shown in Equation (1), a reaction heat of up to 8.67 MJ is released by a unit mass of the composites, which is twice as much as that of TNT (4.18 MJ/kg), and the adiabatic reaction temperature can even reach 3580 K [5].

In recent years, notable progress has been made on the mechanical and reaction properties of PTFE/Al composites. Feng et al. [6] conducted numerous quasi-static compression experiments to research the mechanical properties of PTFE/Al composites, and observed a violent reaction phenomenon for the first time. Then, they investigated the influences of sintering temperature, component ratio, and Al particle size on the mechanical and reactive properties of PTFE/Al through quasi-static compressive tests, and proposed a crack-induced initiation mechanism [7]. Casem performed both quasi-static and dynamic compressive tests of PTFE/Al with a servo-hydraulic universal testing machine and split-Hopkinson pressure bar (SHPB) system, and obtained the stress-strain data, which was used to determine the parameters of the Johnson–Cook model for the material [8]. Ames [9], McGregor [10], and Ge et al. [11] studied the energy-release characteristics of the PTFE/Al mixtures under high speed impact conditions. In addition, researchers also added tungsten (W) to the PTFE/Al composites to improve the material’s strength and density. Up until now, many related studies have been conducted to investigate the material preparation technology [12], mechanical properties [13,14,15,16], and reaction and energy-release properties [17,18] of PTFE/Al/W composites. Moreover, many experimental methods, such as drop-weight apparatus [16,19], scanning electron microscopy (SEM) [7,20,21], X-ray diffraction (XRD) [20,22], and differential scanning calorimetry (DSC) [23,24], have been employed by researchers to conduct further studies.

As is known, titanium hydride (TiH_2_) is a novel potential energetic material with a large storage capacity for hydrogen. When heated to a certain temperature, hydrogen will be rapidly released from TiH_2_. Since hydrogen has an extremely high energy density, a remarkable amount of heat can be produced if TiH_2_ fully released its energy during the reaction. As was reported in reference [25], TiH_2_ possessed a high heat value of 21.5 MJ/kg with 3.9 wt % content of hydrogen, which was far outweighs that of TNT (4.18 MJ/kg) and PTFE/Al (8.67 MJ/kg). Meanwhile, it is stable enough to coexist with potassium perchlorate for 20 years with almost no decomposition [26]. All of these advantages make TiH_2_ a promising high-energy additive to energetic materials. Xue et al. [27] and Cheng et al. [28] introduced TiH_2_ into explosives and found the explosive/TiH_2_ mixtures have a better performance on the explosive parameters; the positive time, the peak overpressure, and the specific impulse of the shock wave were all obviously improved. Li [25] introduced TiH_2_ to the propellant, and found that TiH_2_ would contribute to accelerate the combustion rate of the propellant.

Recently, our research group has added TiH_2_ to the PTFE/Al composite for the first time and conducted a series of quasi-static compression experiments [29]. The results show that TiH_2_ particles help to improve the material’s strength within a certain amount, and they could be activated by the heat produced by the reaction between PTFE and Al, releasing hydrogen and generating titanium carbide (TiC), which makes TiH_2_ completely release its energy. However, there are no reports concerning the mechanical and reaction characteristics of the novel PTFE/Al/TiH_2_ composites under dynamic compression, which is of great importance for the further application of these materials.

In this work, PTFE/Al/TiH_2_ reactive materials with different contents of TiH_2_ are prepared. The reaction energy of the materials is measured by an adiabatic bomb calorimeter, and the reaction processes are analyzed based on the XRD patterns of the reaction products. SHPB tests are then conducted to explore the effects of TiH_2_ content on the dynamic mechanical properties of the composites, and the microstructure of the samples before and after the SHPB tests are observed by SEM. Finally, drop-weight tests are performed to study the impact sensitivity and reaction characteristics of the composites, during which the reaction processes are recorded by a high-speed photography system.

## 2. Materials and Experiments

### 2.1. Material Fabrication

In this study, five kinds of PTFE/Al/TiH_2_ composites were fabricated through a process including powder mixing, cold pressing, and vacuum sintering. The relative mass ratio of PTFE to Al for all composites was determined through zero-oxygen-balance, fixing the mass ratio at 73.5/26.5, and different content of TiH_2_ particles were added as high-energy additives. Table 1 shows the mass fraction of the five composites, along with the theoretical material density (TMD), the corresponding actual density, and relative density.

**Figure 1:**
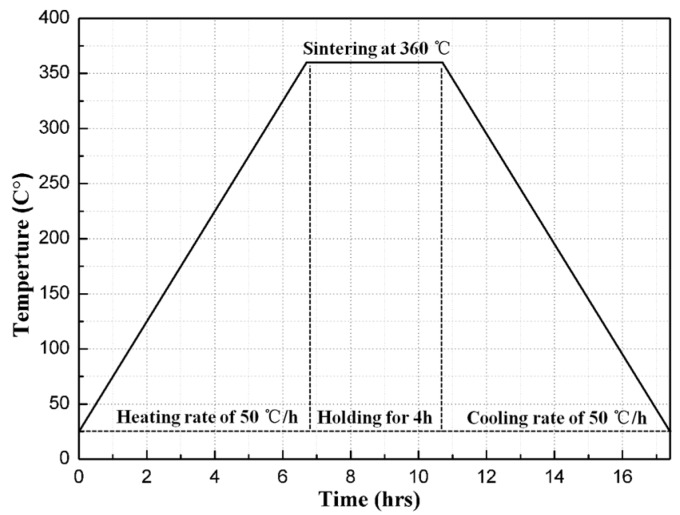
The temperature history of the sintering cycle.

**Figure 2:**
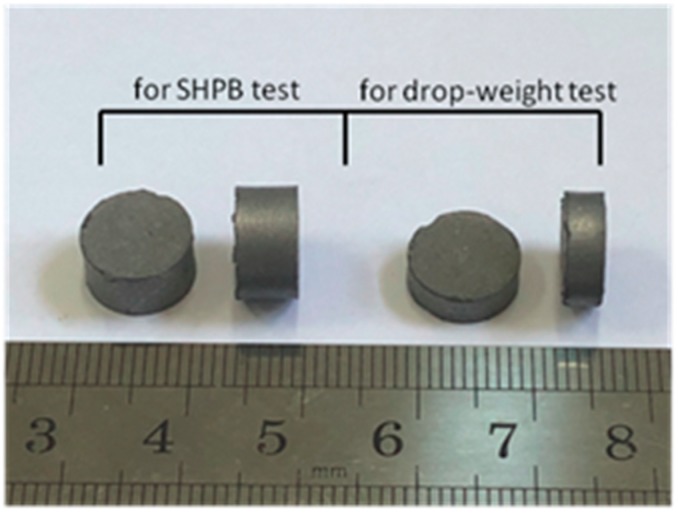
The cylindrical samples after sintered.

The fabrication process was based on Nielson et al.’s [12] patent, but followed a different sintering history. Firstly, the original powders of PTFE (25 μm, 3M, Shanghai, China), Al (1–2 μm, JT-4, Changsha, China) and TiH_2_ (4–6 μm, ZN, Zhuzhou, China) were added to an anhydrous ethanol solution and mixed by a motor-driven blender for approximately 30 min, followed by a drying process at 60 °C for 48 h in a DZG-6050 vacuum drier (SX, Shanghai, China). Then, the dried mixtures were put into a cylindrical mold and cold uniaxial pressed at the pressure of 240 MPa for 2 min, through which the cylindrical specimens were made with the dimension of ϕ10 mm × 5 mm and ϕ10 mm × 3 mm for SHPB and drop-weight tests, respectively. Finally, the cylindrical specimens were vacuum sintered at a temperature of 360 °C. Figure 1 depicted the time history of the sintering cycle. Briefly speaking, it included a heating process of the samples with a rate of 50 °C/h to a final temperature of 360 °C, holding for 4 h, and then cooling to room temperature at the rate of 50 °C/h. The sintered specimens are shown in Figure 2.

**Figure 3:**
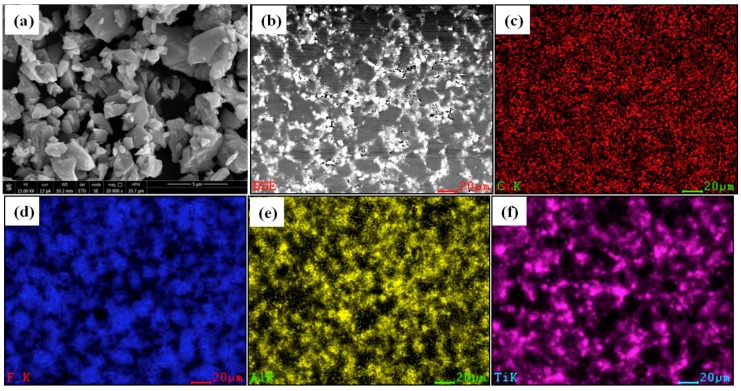
Microstructure and element distributions of the materials. (**a**) Scanning electron microscopy (SEM) image of pure TiH_2_ powder; (**b**) backscattered SEM (BSE) image of PTFE/Al/TiH_2_ (66.2/23.8/10); (**c**) C element; (**d**) F element; (**e**) Al element; and (**f**) Ti element.

In order to evaluate the mixing uniformity of the three components, the original microstructures of the prepared mixtures were observed using an FEI Versa 3D Scanning Electron Microscope (Hillsboro, OR, USA) before the experiments, as is shown in Figure 3. Figure 3a shows the micrograph of pure TiH_2_ particles as received, indicating that the TiH_2_ granules had irregular shapes and sharp edges with the average sizes of 4–6 μm. In this part, the backscattered scanning electron microscopy (BSE) image of PTFE/Al/TiH_2_ (66.2/23.8/10) composite is depicted as an example, as is shown in Figure 3b, in which TiH_2_ granules were relatively bright for the higher atomic number of Ti element, followed by the brightness of Al particles. It is likely that the three components were uniformly mixed, Al and TiH_2_ granules were uniformly distributed in the continuous matrix formed by PTFE. Figure 3c–f shows the distribution of C, F, Al, and Ti elements corresponding to Figure 3b, which further illustrates the homogeneity of the materials.

### 2.2. Measurement of Reaction Energy

An adiabatic bomb calorimeter (Parr 6300, Parr Inc., Moline, IL, USA) was used to measure the reaction energy of PTFE/Al/TiH_2_ under an oxygen atmosphere. Before the measurement, the heat capacity of the calorimeter was calibrated by burning benzoic acid in an oxygen environment with the pressure set at 3 MPa. Then the reaction mechanism was explored based on the XRD (D8 Advance, Bruker AXS Inc., Dresden, Germany) patterns of the reaction products.

### 2.3. Dynamic Compression Tests

**Figure 4:**
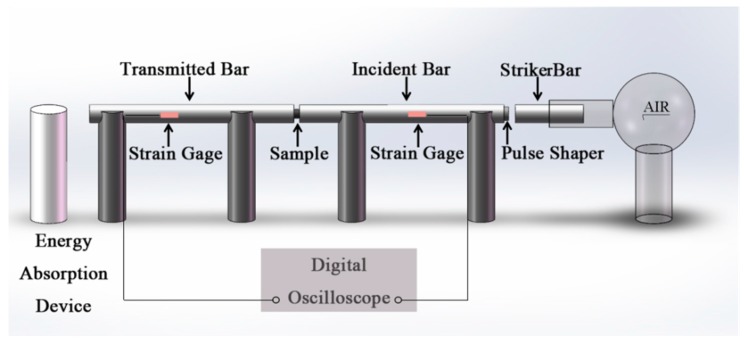
Schematic illustration of the split-Hopkinson pressure bar (SHPB) facility.

A split-Hopkinson pressure bar system (ϕ-20 SHPB, AOC, Hefei, China) was adopted to determine the dynamic mechanical performance of the five composites, which included the striker bar, the incident bar, and the transmitted bar, as illustrated schematically in Figure 4. In this work, the lengths of the three bars were 600 mm, 6000 mm, and 3500 mm, respectively, with the same diameter of 20 mm. Normally, the three bars were made of 6061-T6 aluminum or 440-HT stainless steel. Considering the low impedance of the PTFE/Al/TiH_2_ composites, the lower-modulus aluminum bars were adopted to achieve a stronger transmission signal. In addition, in order to achieve an early uniform uniaxial stress state in the samples, a slowly rising incident wave pulse was required, which was realized by using a pulse shaping technique. In this work, rubber shims with a diameter of 8 mm and a thickness of 1 mm were placed at the front end of the incident bar during impact, the incident wave pulse with a longer rising edge time could be obtained, which may facilitate the state of dynamic stress equilibrium in the specimens [16]. Tests were performed at the temperature of 12 °C, and the ends of the specimens were slightly lubricated with petroleum jelly to minimize the influence of friction restraint.

### 2.4. Drop-Weight Tests

The impact sensitivities and impact-induced reaction properties of the materials were studied by using a drop-weight machine (HGZ-1, TD, Xiangfan, China). The apparatus had a drop hammer with a mass of 10 kg, and it permitted variance of the drop-weight from 0 to 156 cm in 0.5 cm increments. The impact sensitivity of the five PTFE/Al/TiH_2_ composites was determined by the drop height that would trigger the reaction with a 50 percent probability (*H*_50_), and the standard Bruceton test method was adopted to obtain the 50 percent point [30]. During the tests, the samples were directly impacted by the drop mass falling from different heights, and a Phantom V710 high-speed camera with a frame rate of up to 20,000 frames/s (Vision Research, Inc., Wayne, NJ, USA) was employed to capture the reaction phenomena of the samples.

In this work, 26 samples were fabricated for each type. Among them, 25 samples were tested to determine the *H*_50_ of the materials, and the remaining samples were tested at the same drop height to compare the reaction degree. All tests were conducted at an ambient temperature of 25 °C.

## 3. Results and Discussion

### 3.1. Reaction Energy

Table 2 lists the reaction energy per unit mass of the five types of PTFE/Al/TiH_2_ composites under an oxygen atmosphere. From Table 2, it can be observed that the reaction energy of the five composites increased along with the increase of TiH_2_ content, indicating a positive effect of TiH_2_ on the total energy of the reaction system. The reaction heat of the type E composite was determined experimentally to be 16.15 MJ/kg, 3.7 times bigger than that of TNT, which is reported to be 4.32 MJ/kg. Besides, the theoretical reaction heat of PTFE/Al in a vacuum is 8.67 MJ/kg [4], which is much lower than that of the experimental value of type A composite (13.81 MJ/kg) in oxygen, implying the participation of oxygen in the reaction of the PTFE/Al/TiH_2_ composites.

**Figure 5:**
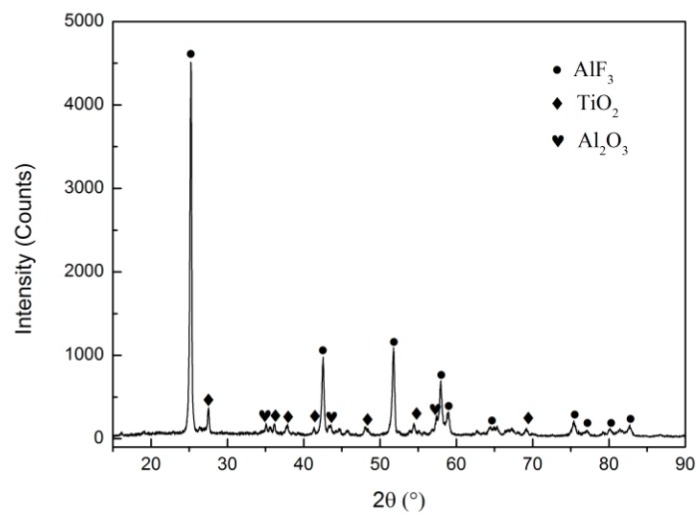
X-ray diffraction (XRD) pattern of the reaction residues in oxygen of type B composite.

In addition, the recovered reaction residues of a type B composite in oxygen was analyzed by X-ray diffraction to explore the chemical reaction mechanism, as is shown in Figure 5. The results indicate that the reaction products were proven to be AlF_3_, TiO_2_ and Al_2_O_3_. Among them, AlF_3_ was the main product, which was produced by the reaction between Al and gaseous C_2_F_4_ releasing from the decomposition of PTFE, accompanied by the production of C (carbon black) (Equations (2) and (3)). However, the color of the recovered reaction residues was white, demonstrating that C had reacted with oxygen to generate CO_2_ or CO in the presence of oxygen (Equations (4) and (5)). A small amount of Al_2_O_3_ was also formed from the oxidation of Al by oxygen (Equation (6)). Meanwhile, TiH_2_ was decomposed when subjected to reaction heat, generating Ti and H_2_ (Equation (7)), which could be oxidized by oxygen to produce TiO_2_ and water vapor (H_2_O) (Equations (8) and (9)), respectively. Furthermore, it is probable for TiF_4_ to be generated from the fluorination of titanium (Equation (10)), but the corresponding diffraction peak could not be detected in the XRD patterns, due to its volatilization [23]. According to the analysis above, the main reaction processes of the PTFE/Al/TiH_2_ composites at oxygen atmosphere can be described by the following equations:

(–C_2_F_4_–) → C_2_F_4_ (g) (2)

4Al + 3C_2_F_4_ → 4AlF_3_ + 6C (3)

C + O_2_ → CO_2_ (4)

C + O_2_ → 2CO (5)

4Al + 3O_2_ → 2Al_2_O_3_ (6)

TiH_2_ → Ti + H_2_ (7)

Ti + O_2_ → TiO_2_ (8)

2H_2_ + O_2_ → 2H_2_O (g) (9)

Ti + C_2_F_4_ →TiF_4_ (g) + 2C (10)

### 3.2. Dynamic Compression Properties

**Figure 6:**
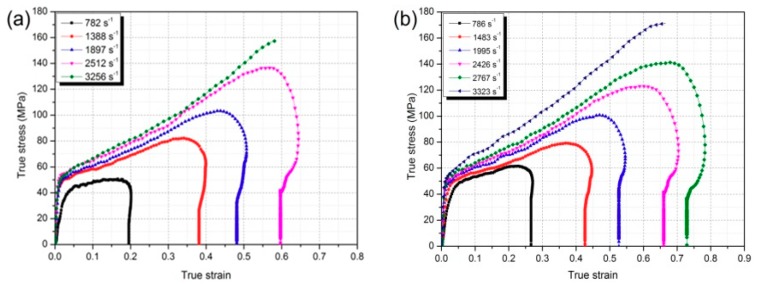

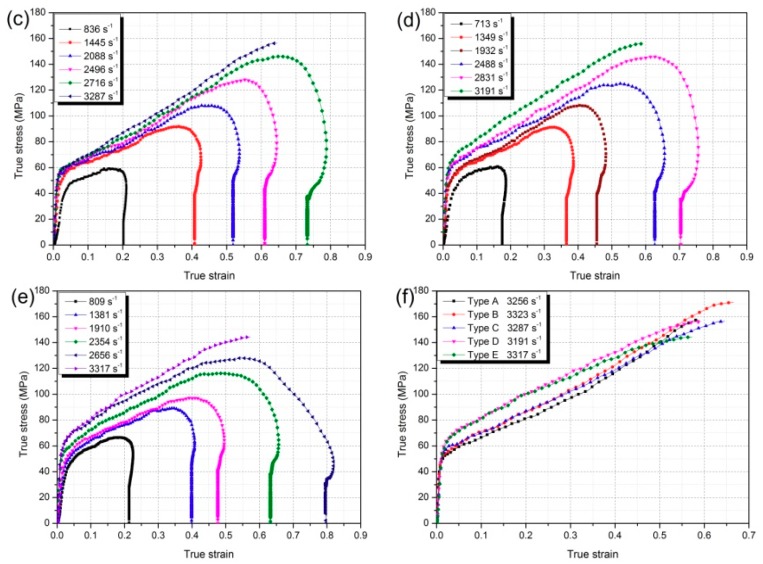
True stress-strain curves of (**a**) type A; (**b**) type B; (**c**) type C; (**d**) type D; (**e**) type E composites at different strain rates, and (**f**) the comparison of the five materials’ mechanical properties at the same strain rate of about 3200 s^−1^.

The dynamic mechanical responses of the five kinds of PTFE/Al/TiH_2_ were obtained through dynamic compression tests, as is shown in Figure 6. From Figure 6a–e, it can be found that all the five composites showed significant strain hardening and strain rate hardening effects, and with the increase of loading strain rates, the yield compressive strength and the hardening modulus showed an increasing tendency. Figure 6f depicts the comparison of the mechanical performance of the five materials at the same strain rate of about 3200 s^−1^, and Table 3 lists the corresponding mechanical parameters. As is shown in Table 3, the yield strength of the materials showed an increasing trend as the content of TiH_2_ increased, and both the hardening modulus and the failure strain were firstly increased and then decreased, indicating that the amount of TiH_2_ had a significant influence on the materials’ mechanical properties. In addition, a certain amount of TiH_2_ helped to increase the material’s compressive strength due to the irregular shapes of the TiH_2_ particles, and the maximum could even reach 173.2 MPa with 5% TiH_2_, which was 10.1% higher than that of PTFE/Al. However, as the content of TiH_2_ reached 30%, the material strength was lower than the PTFE/Al composite. The analysis holds that excessive TiH_2_ granules may destroy the continuity of the PTFE matrix, thus leading to the decrease of the material’s ultimate strength.

**Figure 7:**
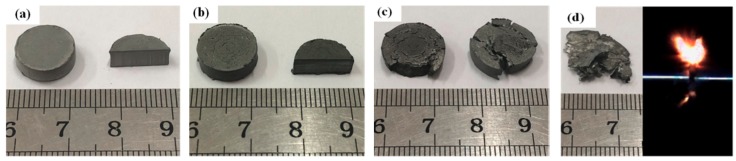
Recovered type B samples after dynamic compression tests at different strain rates: (**a**) 786 s^−1^; (**b**) 1995 s^−1^; (**c**) 2767 s^−1^; (**d**) 3323 s^−1^.

Figure 7 shows the recovered type B specimens after dynamic compression tests at varying strain rates. As is shown in Figure 7a, when the strain rate was relatively low (786 s^−1^), only plastic deformation took place in the specimens, but no fractures appeared in both the surface and interior of the specimens. As the strain rate increased to 1995 s^−1^, many little cracks arose in the outer surfaces, but not in the interior of the specimens (Figure 7b). With further raising of the strain rates, fractures occurred in the tested specimens at a strain rate of 2767 s^−1^ (Figure 7c), and flames could even be observed during the dynamic compression process, at a strain rate of 3323 s^−1^ (Figure 7d), indicating that the reaction happened under a high impact loading rate.

**Figure 8:**
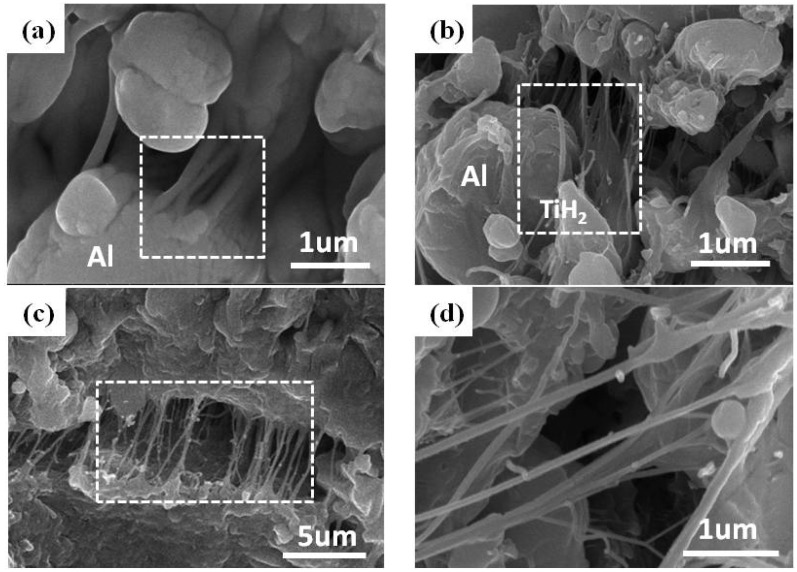
SEM micrographs of type B samples: (**a**) the interior structure before SHPB tests; (**b**) the separation of Al/TiH_2_ particles from the PTFE matrix after SHPB tests; (**c**) fracture of the PTFE matrix after the impact; (**d**) network of PTFE nano-fibers.

Figure 8 shows SEM images of the interior structure of the type B samples before and after the SHPB tests. From Figure 8a, it can be observed that the combination between the aluminum granules and the PTFE matrix was quite close before the SHPB tests, and some discontinuous thick PTFE fibers were also found to enhance the connection. However, after the SHPB tests, Al and TiH_2_ particles were separated from the PTFE matrix, and cracks could also be observed in the PTFE matrix, as shown in Figure 8b,c. Meanwhile, as the PTFE matrix was stretched, many fibers were generated along the stretching direction, and they interwove with each other to form a network structure, which would provide resistance against the propagation of the crack. Figure 8d depicts an enlarged view of these fibers, implying that the fibers are as narrow as 50–200 nm in diameter. Analysis shows that the material failure was mainly caused by the fracture of the PTFE matrix, as well as interface debonding between the reinforcing particles and the PTFE matrix, which is also proposed by other researchers [13,31].

### 3.3. Impact Sensitivity and Reaction Properties

In this research, the standard Bruceton test method, which also called the “up-and-down technique” [30], was adopted to determine the impact sensitivities of the five composites. The characteristic drop height of impact sensitivity (*H*_50_) can be obtained by the Equation (11):

(11) $$H_{50}=A+B\left[\frac{\sum iC_{i}}{D}-\frac{1}{2}\right]$$

where *A* is the lowest height in the test, *B* is the increment of height, *D* is the total number of reaction events in the test, *i* is the order of the drop height beginning from 0, and *C_i_* is the number of reaction events at a certain height.

**Figure 9:**
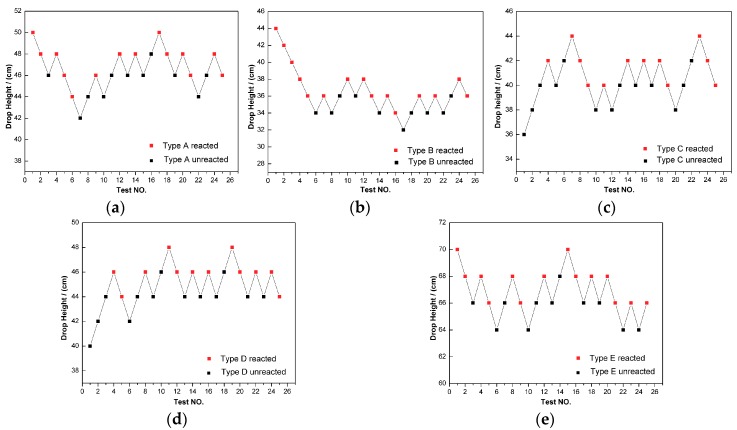
The drop-weight tests data points of the five types of PTFE/Al/TiH_2_ composites: (**a**) type A; (**b**) type B; (**c**) type C; (**d**) type D; (**e**) type E.

Based on the standard Bruceton test method, each drop-height tests data point for the five kinds of PTFE/Al/TiH_2_ is depicted in Figure 9, the corresponding characteristic drop height is tabulated in Table 4. As can be seen from the results, compared with PTFE/Al, the impact sensitivity of the materials is increased as the mass ratio of TiH_2_ increased from 5% to 20%, the reason may be that the irregular shapes of TiH_2_ particles make it easier for the generation of “hot spots” in the PTFE/Al reactive material system. However, when the content of TiH_2_ exceeds 30%, the characteristic drop height of the material increases dramatically, with the *H*_50_ value reaching 66.6 cm, which is 20.2 cm higher than that of the PTFE/Al composites without TiH_2_. As is reported in our previous work [29], the reaction is firstly triggered by the ignition of PTFE and Al in the PTFE/Al/TiH_2_ reactive system, then TiH_2_ is activated by the heat released from the initial reactant. Therefore, excessive TiH_2_ means there is a decreased quantity of the initial reactant and a lower chance of PTFE being located next to Al particles, thus resulting in the enhancement of material insensitivity.

**Figure 10:**
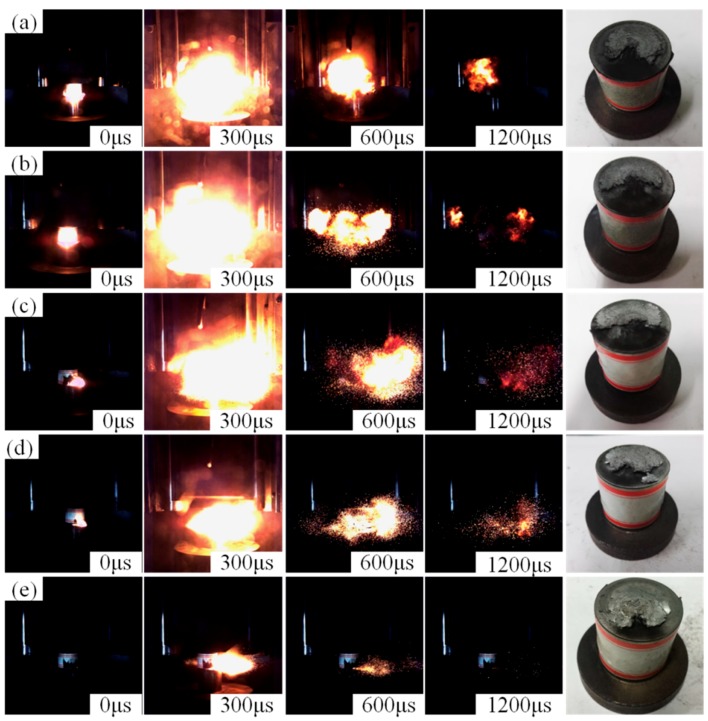
Video sequences of the five PTFE/Al/TiH_2_ composites under drop-weight impact at a height of 90 cm, and corresponding sample residues after the tests: (**a**) type A specimens; (**b**) type B specimens; (**c**) type C specimens; (**d**) type D specimens; and (**e**) type E specimens.

In order to compare the reaction degrees of the five kinds of composites, drop-weight tests were conducted at a certain height of 90 cm. Figure 10 shows video sequences of the reaction phenomena and the corresponding sample residues after the tests. The results indicate that the reaction degree of the composites was significantly affected by the content of TiH_2_. Compared with PTFE/Al, the degree of reaction was improved with the content of TiH_2_ ranging from 5% to 10%, indicating that a proper amount of TiH_2_ contributed to promoting the reaction of PTFE/Al reactive material. When the addition of TiH_2_ was 20%, the reaction degree was similar to that of the PTFE/Al composites. However, as the mass fraction of TiH_2_ reached 30%, the degree of reaction was decreased dramatically, due to the reduced amount of PTFE and Al. Besides, discontinuous and scattering sparks were jetted from the reaction region of the four composites with TiH_2_, but not from the PTFE/Al reaction region. Furthermore, from the recovered sample residues in Figure 10, the reactions of the composites were more and more incomplete with the increase in TiH_2_ mass fraction, and all the reactions were initiated at the open crack near the edge of the samples. As is known, the largest shear stress was mainly localized in the outer edge of the cylindrical specimens during the deformation process [32]; thus, we can speculate that the shear failure in the outer surface of the samples contributed to the initiation of the materials, indicating the applicability of the shear-induced initiation mechanism in these types of materials [1,9].

## 4. Conclusions

In this paper, TiH_2_ is introduced into PTFE/Al reactive materials for the first time, and its effects on the reaction energy, impact sensitivity, mechanical, and reaction properties of the five types of PTFE/Al/TiH_2_ composites are investigated. The conclusions can be drawn as follows:

(1) With an increase of TiH_2_ content, the reaction energy of PTFE/Al/TiH_2_ under an oxygen atmosphere obviously increases. Especially, the reaction heat of type E composites reaches up to 16.15 MJ/kg, which is 3.7 times than that of TNT.
(2) All the five types of composites show strain hardening and strain rate hardening effects; the yield strength and hardening modulus increases with the increase of strain rates. A relatively low mass ratio of TiH_2_ granules help to improve the materials’ compressive strength, and the maximum even reaches 173.2 MPa with a 5% TiH_2_ percentage, which is 10.1% higher than that of PTFE/Al. Excessive TiH_2_ granules would lead to a decrease of the material’s strength. SEM images of the recovered samples indicate that the fracture of the PTFE matrix, and interface debonding between the reinforcing granules and PTFE matrix are the main mechanisms for material failure.
(3) With the increase of TiH_2_ content from 5% up to 30%, the material’s impact sensitivity shows a decreasing trend. Compared with PTFE/Al, the addition of TiH_2_ (less than 20%) would enhance the impact sensitivity of the materials, while excessive TiH_2_ (more than 30%) would significantly reduce the material’s sensitivity.
(4) The material’s reaction degree is sensitive to the mass ratio of TiH_2_. At a certain drop height of 90cm, the reaction degree of the materials becomes stronger first and then weaker with an increase of TiH_2_ content, which would be the most violent at a 5% content of TiH_2_. In addition, special sparks fly off from the reaction zone of the four composites with the TiH_2_ particles, but not from the PTFE/Al reaction zone. The recovered sample residues indicate that the reaction proceeds more and more incompletely with an increased TiH_2_ mass fraction, and a shear-induced initiation mechanism is applicable to these types of reactive materials.

## Figures and Tables

**Table 1:** Components and theoretical material density (TMD) of the polytetrafluoroethylene (PTFE)/Al/TiH_2_ granular composites.

Type	Mass Fraction (wt %)			TMD (g cm^−3^)	Density (g cm^−3^)	Relative Density
	PTFE	Al	TiH_2_			
A	73.5	26.5	0	2.31	2.20	95.2%
B	69.8	25.2	5	2.36	2.24	94.9%
C	66.2	23.8	10	2.41	2.33	96.7%
D	58.8	21.2	20	2.52	2.42	96.0%
E	51.5	18.5	30	2.63	2.51	95.4%

**Table 2:** Reaction energy of the PTFE/Al/TiH_2_ composites in an oxygen atmosphere.

Type	Reaction Energy (MJ/kg)
A	13.81
B	14.39
C	14.88
D	15.53
E	16.15

**Table 3:** Mechanical parameters of PTFE/Al/TiH_2_ composites under dynamic compression loading.

Type	Yield Strength (MPa)	Hardening Modulus (MPa)	Ultimate Strength (MPa)	Critical Failure Strain
A	49.6	189.3	157.3	0.58
B	52.3	192.4	173.2	0.66
C	58.8	172.1	156.5	0.65
D	63.3	164.3	156.1	0.59
E	65.7	144.7	142.8	0.57

**Table 4:** The characteristic drop height of impact sensitivity (*H*_50_) for the five types of PTFE/Al/TiH_2_ composites.

Type	Characteristic Drop Height (cm)
A	46.4
B	36.6
C	40.7
D	45.0
E	66.6
